# Identifying Calcaneal Anatomical Regions of Interest (ROI) for Quantitative Ultrasound Application in Subadults

**DOI:** 10.1007/s00223-022-01018-3

**Published:** 2022-08-31

**Authors:** Connor S. Blythe, Mikaela S. Reynolds, Laura S. Gregory

**Affiliations:** Clinical Anatomy and Paediatric Imaging Research Laboratory, School of Biomedical Sciences, Faculty of Health, Queensland University of Technology, Brisbane, Queensland Australia

**Keywords:** Calcaneus, Region of interest, Computed tomography, Quantitative ultrasound, Pediatric development

## Abstract

Whilst quantitative ultrasound can be reliably used to assess bone health in adults, the fixed location of the transducers in current devices may result in inaccurate and unreliable measurements in bone assessment in children due to the variation in foot size during growth. To improve positioning for paediatric assessment, Jaworski et al. (1995) created an anatomical method to identify the region of interest (ROI), however, there have been no medical imaging studies to confirm that the Jaworski method results in consistent placement of the transducer on the centre of the calcaneal body to avoid edge artefacts. In this study, computed tomography scans of the tarsus were collected from 498 individuals (258 females; 240 males) aged 2 to 20 years and used to create three novel anatomical methods to identify ROI on the calcaneus using palpable landmarks. In addition, the established Jaworski method was applied to the same scans and compared to our novel methods. The maximum ROI significantly increased with age with males having significantly greater diameters, supporting the recommendation that ½ inch diameter transducers should be used on individuals younger than 7 years of age. We identified that 79% of the ‘Jaworski points’ lied anterosuperior to the ROI centre point identified in this study, with 10% of the points lying outside the ROI. Of the three novel methods, only the calcaneal insertion method demonstrated small measurement variance between individuals of the same age in each sex and is therefore the preferred method for ultrasound clinical application.

## Introduction

Quantitative ultrasound (QUS) and its parameters including broadband ultrasound attenuation (BUA in dB/MHz) and speed of sound (SOS in m/sec), have been proposed as an alternative method of assessing bone quality as it is non-ionising and portable compared to the current gold-standard dual-energy x-ray absorptiometry (DXA), which is currently used most often in the clinical assessment of bone volume fraction and mineral content [1]. Commercial QUS scanners such as the GE Lunar Achilles Densitometer, Hologic Sahara, Ubis 3000, and Med-Tec QUS2 have been developed for bone quality assessment of the calcaneus in adults using fixed in-place one inch (25.4 mm) transducers relative to a footplate [2]. In adults the use of these fixed locations has demonstrated relatively good accuracy and precision due to large calcanei [3–5]. However, individual variations in foot and calcaneal morphology can lead to the fixed ROI to be located at a different anatomical region, which may have a significantly different bone density and structure [6–10].

Although QUS was initially designed for adults, it has been performed in paediatric populations for the assessment of bone health using the calcaneus, phalanges of the hand, and the patella [10–21]. Application in subadult populations however, may result in an ROI located outside of the recommended region on the posterior aspect of the calcaneus, which may lead to the inclusion of soft tissue or cortical bone edges due to the fixed location of the transducers relative to the footplate [8, 10, 11]. Inaccurate ROI placement can lead to poor correlations with DXA measurements as bone mineral content varies according to the anatomical region investigated [6]. In addition to this, most commercial systems use 25 mm diameter ultrasound transducers due to the relatively large adult calcanei, however, the use of this size in subadult populations may result in the inclusion of surrounding soft tissue and cortical bone edges which will lead to inaccurate measurements of bone quality [2, 6, 22].

To date, two studies have proposed methods to identify anatomical ROIs from palpable landmarks to reduce variation in the placement of ultrasound transducers [8, 9]. From these studies only Jaworski et al. (1995) has investigated an anatomical ROI from palpable landmarks in a paediatric population, with their proposed method having comparable precision to that of adults due to changes or adaptions being made to the machine or paediatric foot position. These adaptions include the use of footpads to position smaller feet and reducing the ultrasound transducer from 25 to 10 mm in diameter to minimise contact with surrounding structures. Regardless of these adaptions, the proposed method by Jaworski et al. (1995) has not been validated using medical imaging and therefore the resultant anatomical location may not reside within the target region in all subadult ages due to accelerated growth of the calcaneus.

Therefore, the location of the most suitable anatomical region of interest for a paediatric population for ultrasound transducer placement that will avoid cortical bone or surrounding soft tissue interference is still not known. In this study we analysed a large computed tomography subadult dataset to investigate the effect of age and sex on calcaneal region of interest size, and compare a number of novel methodologies introduced in this study to the Jaworski et al. (1995) method to improve transducer placement in quantitative ultrasound application in subadults for bone quality assessment.

## Materials and Methods

**Fig. 1:**
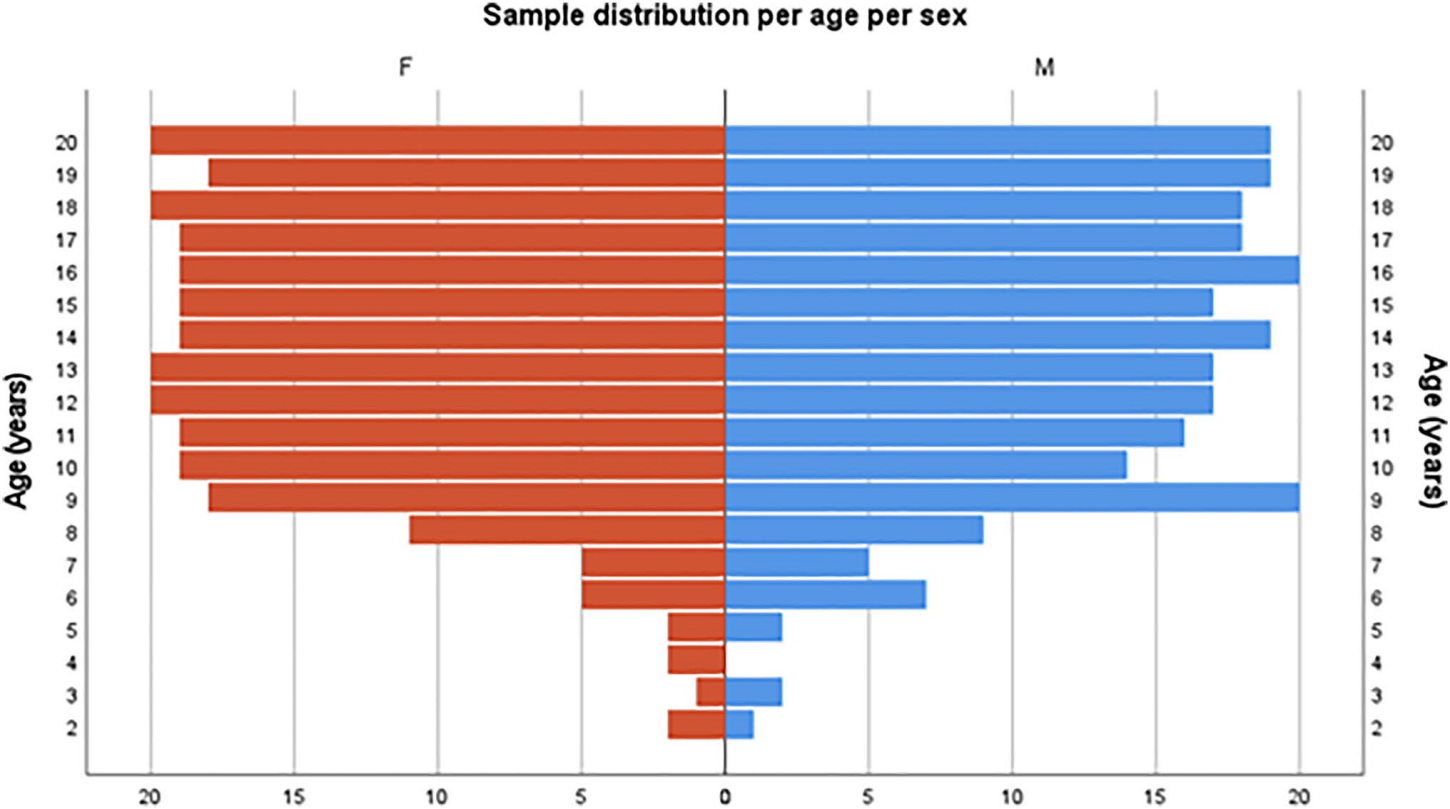
Frequency distribution of computed tomography (CT) samples per year of age per sex (F/red = female; M/blue = male)

The study sample consisted of 498 retrospective multi-slice computed tomography (MSCT) scans (258 females, 240 males) of the calcaneus of patients aged two to 20 years. All individual scans were collected by a radiologist from the Queensland Health Enterprise PACS database, representing North-Eastern hospitals in Australia. As all samples were collected retrospectively, limited CT scans were available from young children as seen in Fig. 1. All scans were conducted between 2010 and 2020 at a maximum slice thickness of 4 mm with a CT scanning parameter range of 80–120 kV, 25–81 mA, and 4–46 table feed per rotation. Scans were excluded from collection if the radiology report described the presence of any metabolic or skeletal disorders that may affect growth, or trauma such as fractures to the calcaneus. It should be noted that scans were included if patients had fractures to surrounding bones but not to the calcaneus, with approximately 85 patients having traumatic fractures to either the tibia or fibula. At the Queensland Children’s Hospital, DICOM (Digital Imaging and Communications in Medicine) files were imported into OsiriX^TM^ (Version 4.1, 64 bits; Visage Imaging GmbH, San Diego, CA) for deidentification with the metadata including the patient’s date of scan, age, and sex retained. Ethical approval was granted by The Children’s Health Queensland Hospital and Health Service Human Research Ethics Committee (LNR/19/QCHQ/51243), ratified by the Queensland University of Technology Research Ethics Unit (Approval No. 1900000946), and approved by the Queensland Government under the Public Health Act (Section 284) 2020 (RD008018).

### ROI Location and Anatomical ROI Methods

**Fig. 2:**
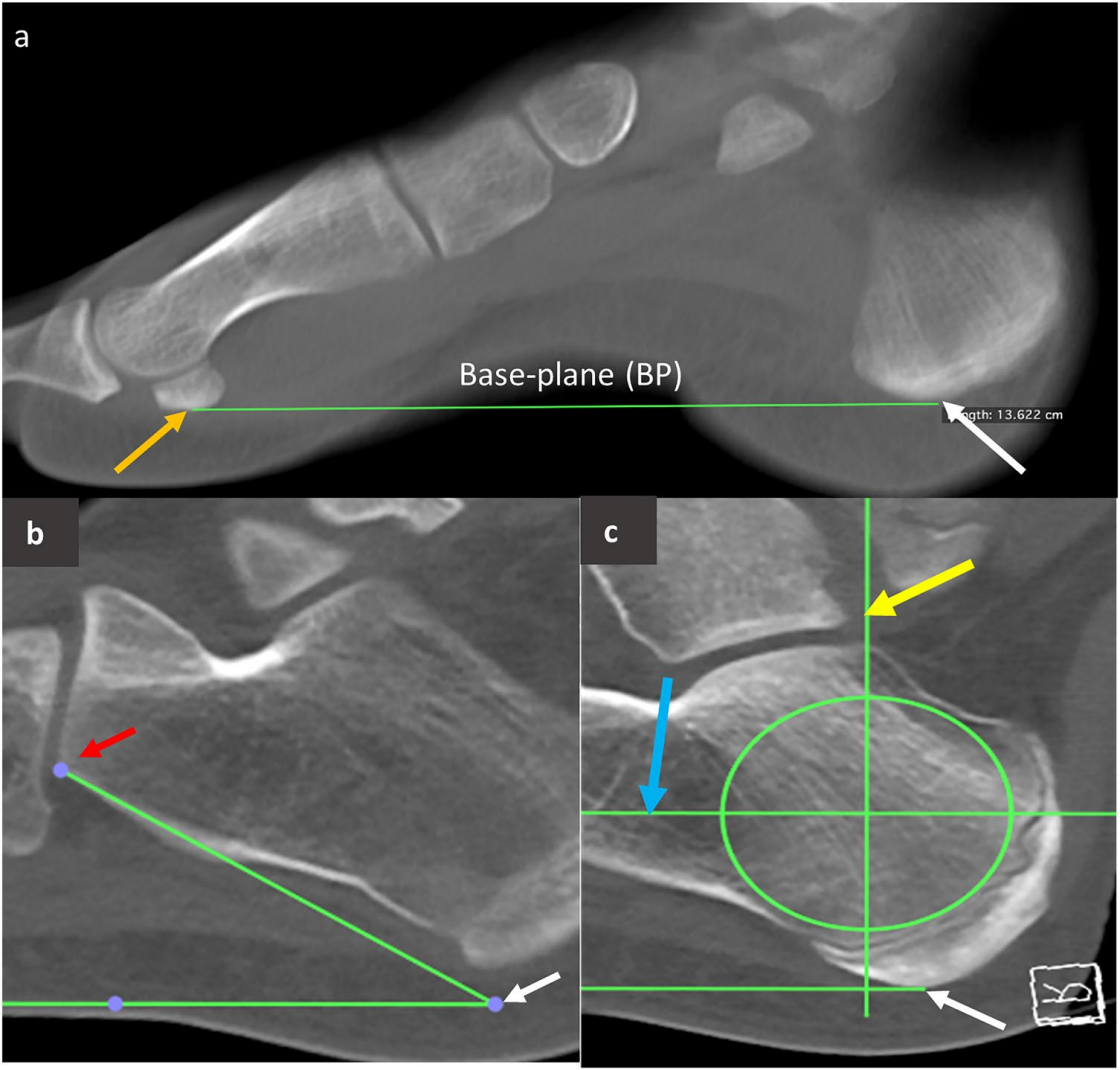
Sagittal MSCT scans of the tarsus demonstrating standardisation of foot position and maximum region of interest selection. **a** Sagittal slice of tarsus, demonstrating the true horizontal base-plane (BP) (green line) from the inferior aspect of the lateral sesamoid bone of the 1st metatarsal (orange arrow) to the most inferior aspect of the calcaneus or calcaneal apophysis (if present) (white arrow). **b** Calcaneal inclination angle is measured from two anatomical landmarks: the inferior aspect of the calcaneal facet for cuboid (red arrow), and the most inferior aspect of the calcaneal apophysis (as indicated by the white arrow, as seen in panel a, b, and c, noting that the inferior aspect of the calcaneal apophysis and the inferior aspect of the calcaneal facet for cuboid cannot be seen in the same sagittal slice). **c** The maximum region of interest circle is drawn to not overlap with the calcaneal apophyseal growth plate, or cortical bone edges of the calcaneus. The centre point of region of interest is then found by two intersecting lines, one parallel to the base-plane horizontally (blue arrow) and one perpendicular to this line vertically (yellow arrow), with the ROI centre point indicated by the intersection of the two lines. Scan was taken from a 10 year-old female

All DICOM data was imported into Horos for quantitative measurements. To standardise the measurements between individuals, a virtual “base-plane” was created which would function as the floor plate, in which the patient’s foot would be ‘standing on’ during clinical application of QUS. This base-plane was created by drawing a horizontal line between the most inferior aspect of the first metatarsal sesamoid bones (if absent, the most inferior aspect of the first metatarsal head was used), and the most inferior aspect of the calcaneal body or calcaneal apophysis, depending on the age of the individual, as seen in Fig. 2a.

The calcaneal inclination angle is used to methodologically standardise the base-plane in cases where the forefoot was not present within the field of view and the first metatarsal sesamoid bones were not present. To measure the calcaneal inclination angle, three points were used: (1) the most inferior point of the calcaneal facet for the cuboid, (2) the most inferior point of the calcaneal body or calcaneal apophysis, and (3) the base-plane created previously, as seen in Fig. 2b.

The location of the maximum region of interest (ROI_max_) and its diameter (mm) was identified by drawing a circle around the largest area of trabecular bone that avoided cortical bone edges of the calcaneus within a sagittal plane through the middle of the calcaneal body (Fig. 2c). This ROI represents the most suitable location for ultrasound transducer placement for accurate QUS assessment of bone quality. To determine which sagittal plane to use, three criteria were used: (1) if the entire foot was present within the field of view, the sagittal plane of the scan was adjusted so it passed through the posterior aspect of the heel and the body of the third metatarsal, (2) the sagittal slice contained the largest posterior height (superior to inferior) of the calcaneus, (3) the insertion of the calcaneal tendon could be observed on the superoposterior aspect of the calcaneus.

**Fig. 3:**
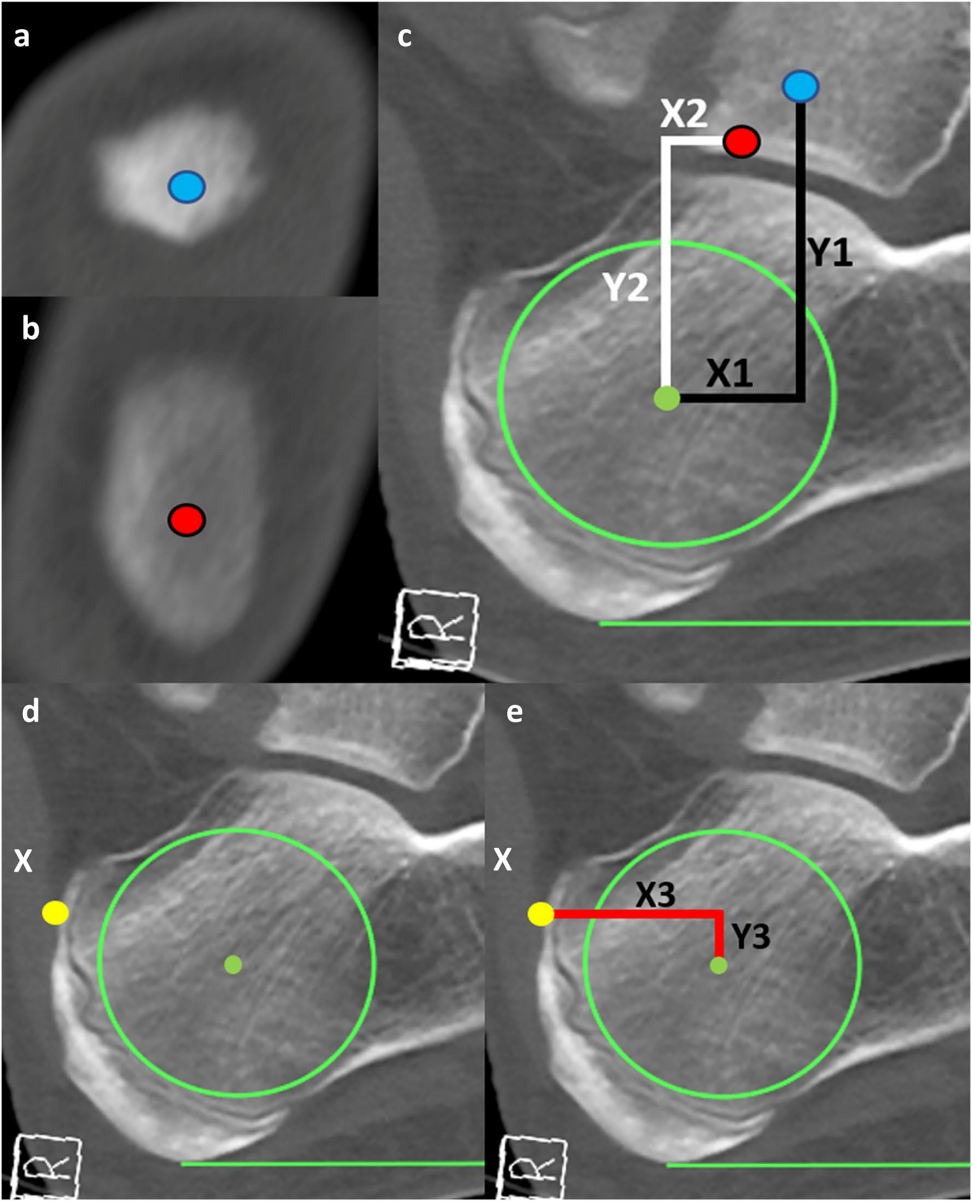
Sagittal MSCT scans depicting coordinate measurements from superficial landmarks to the region of interest centre point (solid green circle). **a** Most medial slice of the medial malleolus and **b** most lateral slice of the lateral malleolus, with the blue and red dot representing the most superficial aspects of each malleolus, respectively. **c** The relative position of each superficial malleolus point is indicated by the blue and red dots which were pasted onto the same midsagittal slice as the region of interest circle. From there vertical (Y1, Y2) and horizontal (X1, X2) lines (black and white lines) were measured from the centre of the region of interest to the medial and lateral malleoli dots. **d** The calcaneal tendon (white X) can be seen, with the most superior aspect of the insertion of the calcaneal tendon identified by the yellow dot. **e** Vertical and horizontal red lines were measured from the centre point of the region of interest to the yellow dot to calculate Y3 and X3

After identifying the centre point of the ROI, vertical and horizontal distances were measured from the centre point to three separate palpable landmarks including the superficial aspects of the lateral and medial malleoli, and the superior aspect of the calcaneal tendon insertion on the calcaneus (inferior to the retrocalcaneal bursa) to create three novel methods (Fig. 3). The calcaneal tendon method was applied to all CT scans, however, due to the retrospective design of this study, the malleoli methods were not applied to 85 CT scans as they contained fractures to the tibia or fibula.

It should be noted that the most superficial aspects of the distal tibial epiphysis and distal fibular epiphysis were used in individuals in which the secondary ossification of the malleoli had not yet commenced; for simplicity, we will refer to these points as the malleoli irrespective of patient age in this paper. In addition, the anatomical ROI method established by Jaworski et al. (1995) was applied to the sagittal CT scans by measuring the distance between the 5th metatarsal tuberosity and the most posterior aspect of the skin of the heel. A point was marked along the line at 1/3rd of the distance from the heel. From there a 1 cm line was drawn superior to this point, perpendicular to the line connecting the posterior heel to the tuberosity to identify the ‘Jaworski point’. Using the Jaworski et al. (1995) anatomical method, the ‘Jaworski point’ was created in 216 individuals where both the hindfoot and midfoot were visible within the field of view, where vertical and horizontal distances were measured from the ‘Jaworski point’ to the ROI centre point created in this study.

Inversion of the subtalar joint was measured using a coronal CT scan, in which a vertical line was drawn through the midline of the diaphysis of the tibia, with that line continuing until it passed through the most inferior aspect of the calcaneus. In the same coronal slice a second line was drawn from the most inferior aspect of the calcaneus to the most superior aspect, making sure that this line intersected the midpoint of the calcaneus. The midpoint was found by measuring the distance between the most lateral and medial aspects of the calcaneus in the coronal slice and identifying the half-way point. The angle tool was used to measure the angle between the two lines. Note that scans that contained fractures to the tibia were not used as they could affect the position of the tibial midline.

Flexion of the talocrural joint was measured in a parasagittal plane or in a pseudo-radiograph which was created by stacking the CT sagittal slices together, so structures over-lied each other and appeared as a lateral radiograph. A linear line was drawn from proximal to distal along the midline of the fibula (depending on if any fractures were present, the tibia may have been used) with the distal end of the line extending past the anterior border of the calcaneus. A second line was drawn longitudinally through the midline of the diaphysis and epiphyses of the 5th metatarsal, making sure that both lines intersected. From there the angle tool was used to measure the angle between the two lines. This approach was used as it virtually simulated the use of a goniometer.

A small number of our retrospectively collected CT scans contained fractures of the tibia, fibula, or tarsal bones, with exception of the calcaneus. For the lateral and medial malleoli methods, scans were excluded if they contained fractures to the distal aspects of the tibia and fibula, however they were not excluded for the remaining methods.

### Statistical Methods

Polynomial and spline models were used to model the non-linear relationship between independent and dependent variables such as age (years), sex, region of interest diameter, and distance measurements from palpable landmarks to the centre of the region of interest. Dependent variables including flexion and inversion of the talocrural and subtalar joints, respectively, were measured in 103 individuals (20.68% of sample) ranging from 2 to 20 years, and then incorporated into the models. Modelling was performed in R Studio (2021, Ghost Orchid) with sex being specifically modelled. Differences between modelling types can be seen below [23].

Polynomial regression: this type of regression adds quadratic or cubic terms to the regression equation to better fit data that is curvilinear in nature.

Spline regression: due to polynomial regressions only being able to capture a certain amount of curvature in a curvilinear relationship, splines can be used to provide a smooth interpolation line between fixed points (knots). In our data set this was done by adjusting or increasing the degrees of freedom.

To accurately model polynomial and spline regressions in our data set, individuals under 5 years of age were grouped together to increase the number of samples in the 2- to 5-year age range.

Using the established anatomical Jaworski et al. (1995) method, the ‘Jaworski point’ was created. A vertical and horizontal distance from the ‘Jaworski point’ to the ROI centre point introduced in this study was measured. An independent samples T-test was used to determine if a sex difference existed. A one-way ANOVA with a Tukey post-hoc test was also used to determine if the distance measurements between these two points changed significantly with age.

## Results

Descriptive statistics including the standard deviation, and minimum and maximum values for the vertical and horizontal distances for the three novel methods and ‘Jaworski’s point’ were calculated using SPSS, version 25 (2015; IBM Corporation, Armonk, NY). From the regression modelling, sex was statistically different in all measurements (*p* < 0.01) and therefore was split for all further analyses. Age was significant in all measurements (*p* < 0.01), however using a post-hoc Tukey test it was noted that neighbouring ages were not statistically significant and age ranges were grouped accordingly.

### Region of Interest

**Fig. 4:**
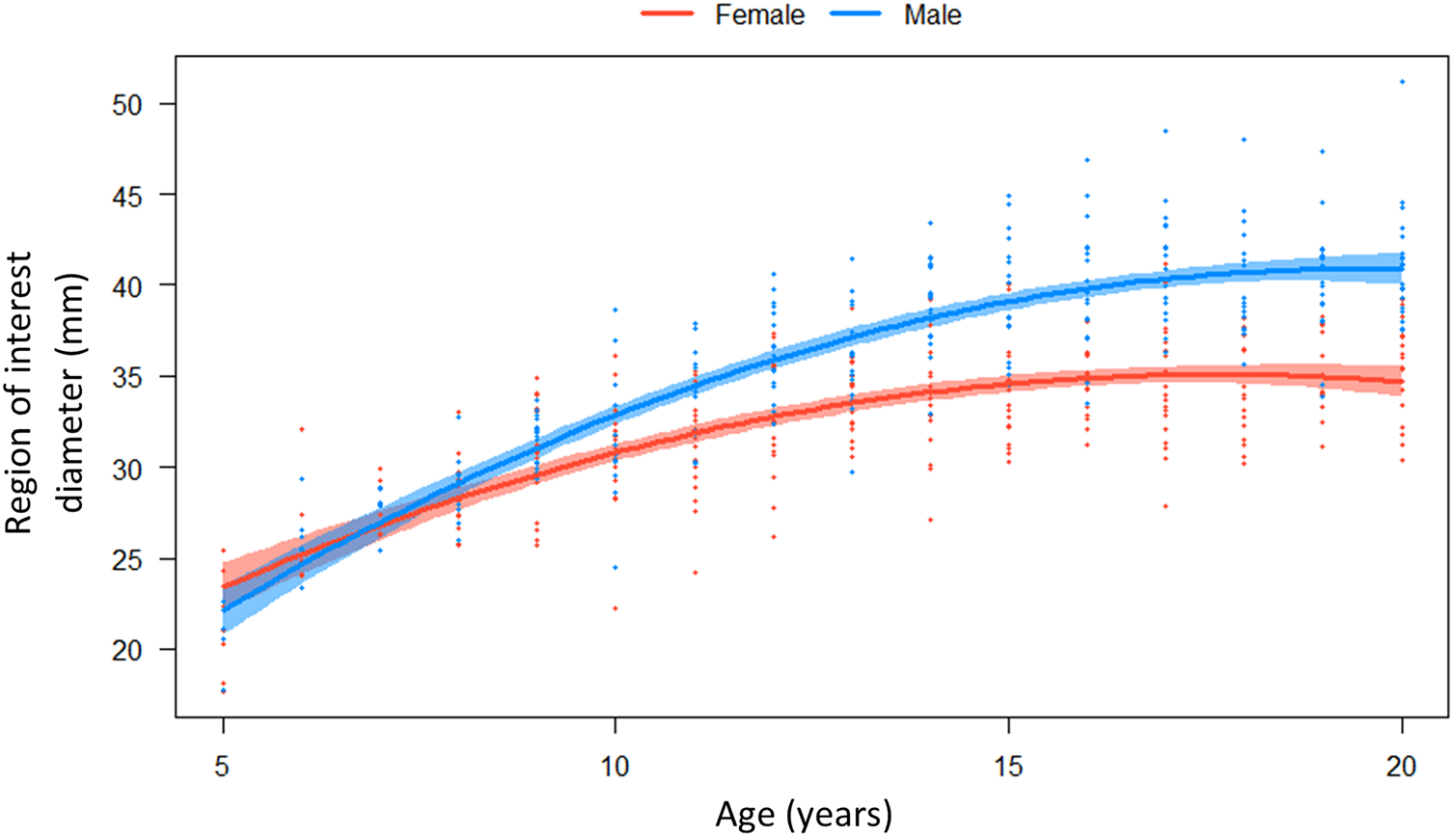
Quadratic regression model for the region of interest diameter (mm), with the red line (female) and blue line (male) representing the mean diameter for each age category, with age and sex having a significant effect. Noting that all samples under the age of 5 years (2 to 5 years) were pooled due to low sample numbers for both females and males

Region of interest diameter (mm) and its relationship with sex and age were best described using a quadratic regression model compared to cubic or spline regressions as the Akaike information criterion (AIC) was lowest at 2436.30. The AIC indicated that a quadratic model fitted the data best at an *R*^2^ of 0.71, *p* < 0.01, with residual standard error of 2.80. The quadratic regression model can be seen in Fig. 4.

A type three analysis of variance (ANOVA) for the quadratic regression model was conducted to explore the impact of age and sex on region of interest size. When investigating the main effects and interactions in this model it was seen that sex, quadratic model for age, and the main interaction between sex and the quadratic model were all significant (*p* < 0.01). There was a statistically significant difference between males and females, *p* < 0.01, with males demonstrating larger ROI diameters in all age categories after 8 years of age. The quadratic model explained significant variability in ROI diameter, *p* < 0.01. The sex * quadratic interaction was also statistically significant, *p* < 0.01.

The smallest region of interest diameter size for both females and males was 17 mm in children aged 2 years. The mean region of interest diameter for each year of age ranged from 23 to 34 mm, and 23 mm to 40 mm for females and males aged 2–20 years, respectively.

### Lateral and Medial Malleoli Methods

The vertical distance measurements from the ROI centre point to both the lateral and medial malleoli were modelled using a cubic regression (lateral AIC = 2753.88, medial AIC = 2914.02), while the horizontal distances for both malleoli were modelled using a quadratic regression (lateral AIC = 2998.24, medial AIC = 3172.12).

The vertical distance for both the lateral and medial malleoli methods with age demonstrated an *R*-squared value of 0.38 (*p* < 0.01) and 0.506 (*p* = < 0.01), respectively. Based on the ANOVA output, the vertical distance to both malleoli demonstrated a significant age effect (*p* < 0.01), with distance increasing with age. There was also a significant sex difference (*p* < 0.01) with male measurements being significantly larger. For females the minimum and maximum vertical distance to the lateral malleolus was 21.22 mm and 48.62 mm with a SD of 4.54 mm, and variance of 20.67 mm; and medial vertical minimum and maximum distances of 26.56 mm and 63.95 mm, SD = 6.20 mm, and variance = 38.48 mm. For males: lateral vertical minimum and maximum distances were 22.83 mm and 50.76 mm, SD = 5.30 mm, and variance = 28.11 mm; and medial vertical minimum and maximum distances were 24.37 mm and 67.97 mm, SD = 7.37 mm, and variance = 54.38 mm.

The horizontal distance for both malleoli methods with age demonstrated an *R*-squared value of 0.248 (*p* < 0.001) and 0.344 (*p* < 0.001), respectively. Based on the ANOVA output, the horizontal distance to both malleoli demonstrated a significant increase with age (*p* < 0.01). Only the lateral horizontal distance demonstrated a sex difference (*p* < 0.01) with male measurements being significantly larger compared to females. For horizontal distances, negative values were provided to points that lied posterior to the ROI centre point, and positive values to points that lied anterior to the ROI centre point. For females: lateral horizontal minimum and maximum distances were − 4.26 mm and 24.86 mm, SD = 5.82 mm, and variance = 33.88 mm; for males: lateral horizontal minimum and maximum distances were − 5.29 mm and 28.99 mm, SD = 5.92 mm, and variance = 28.11 mm. Combined male and female medial horizontal descriptive statistics: minimum and maximum distances were − 5.01 mm and 42.72 mm, with a SD of 8.02 mm and variance of 64.34 mm.

Flexion angle of the talocrural joint and inversion of the subtalar joint were added into all models to determine if they had any significant effect on distance measurements. Flexion angle significantly affected all distance measurements for both malleoli methods (*p* < 0.01). With flexion angle added, the sex effect was no longer significant, with only the horizontal distances for both medial and lateral malleoli increasing significantly with age (*p* < 0.01). Inversion angle of the subtalar joint was significant for all distance measurements except the vertical distance to the medial malleolus. With inversion angle added, similarly there was no longer a sex difference present (*p* > 0.05), with all three distance measurements (lateral vertical distance, medial and lateral horizontal distances) significantly increasing with age (*p* < 0.01).

### Tendon Insertion Method

To model the vertical distance from the insertion of the calcaneal tendon to the centre point of the region of interest, a spline model was chosen with 9 degrees of freedom with an AIC of 1709.78 and a *R*^2^ of 0.7673 (*p* < 0.001). The model had no significant sex effect however, there was a significant sex * age interaction (*p* < 0.01), with males demonstrating larger distances with age compared to females. The minimum and maximum vertical distance was 7.08 mm and 22 mm, respectively, with a standard deviation of 2.78 mm, and variance of 7.76 mm.

For the horizontal distance, a spline model with 8 degrees of freedom was chosen with an AIC of 1865.82 and a *R*^2^ of 0.36 (*p* < 0.01). Like the vertical distance there was no significant sex effect, but a significant sex * age interaction was observed (*p* < 0.01), with males demonstrating larger distances with age compared to females. The minimum and maximum horizontal distance was 1.89 mm and 24.89 mm, respectively, with a standard deviation of 2.18 mm, and variance of 4.76 mm. A summary of the descriptive statistics can be seen in Table 1 and is recommended for use when applying this method in a clinical setting.

**Table 1:** Descriptive clinical table for identification of preferred ultrasound transducer placement on the calcaneus when applying the calcaneal insertion method developed for subadults younger than 20 years of age

Sex	Age sub-groups (years)	Mean ROI diameter (mm)*	Mean vertical distance (mm)	Mean horizontal distance (mm)
Female	5–6	23.45	8.89	16.96
	7–9	30.19	11.74	19.05
	10–11		13.58	19.92
	12–13	34.39	15.08	19.67
	14–20		16.71	18.21
Male	5–6	23.91	9.53	17.58
	7–8	30.57	10.99	18.63
	9–10		12.95	19.99
	11–13	35.78	14.99	20.97
	14–15	40.14	16.54	21.18
	16–17		18.25	19.40
	18		17.22	19.71
	19		18.95	19.35
	20		16.69	21.19

Due to low sample sizes individuals from 2 to 5 years of age were pooled together. Each age subgroup was formed by pooling neighbouring ages which were not statistically different using a Tukey post-hoc test

*Mean regions of interest diameters were pooled for non-significantly different (*p* > 0.05) neighbouring age categories based on a Tukey post-hoc test

### Applicability of Jaworski et al. (1995) Method

**Fig. 5:**
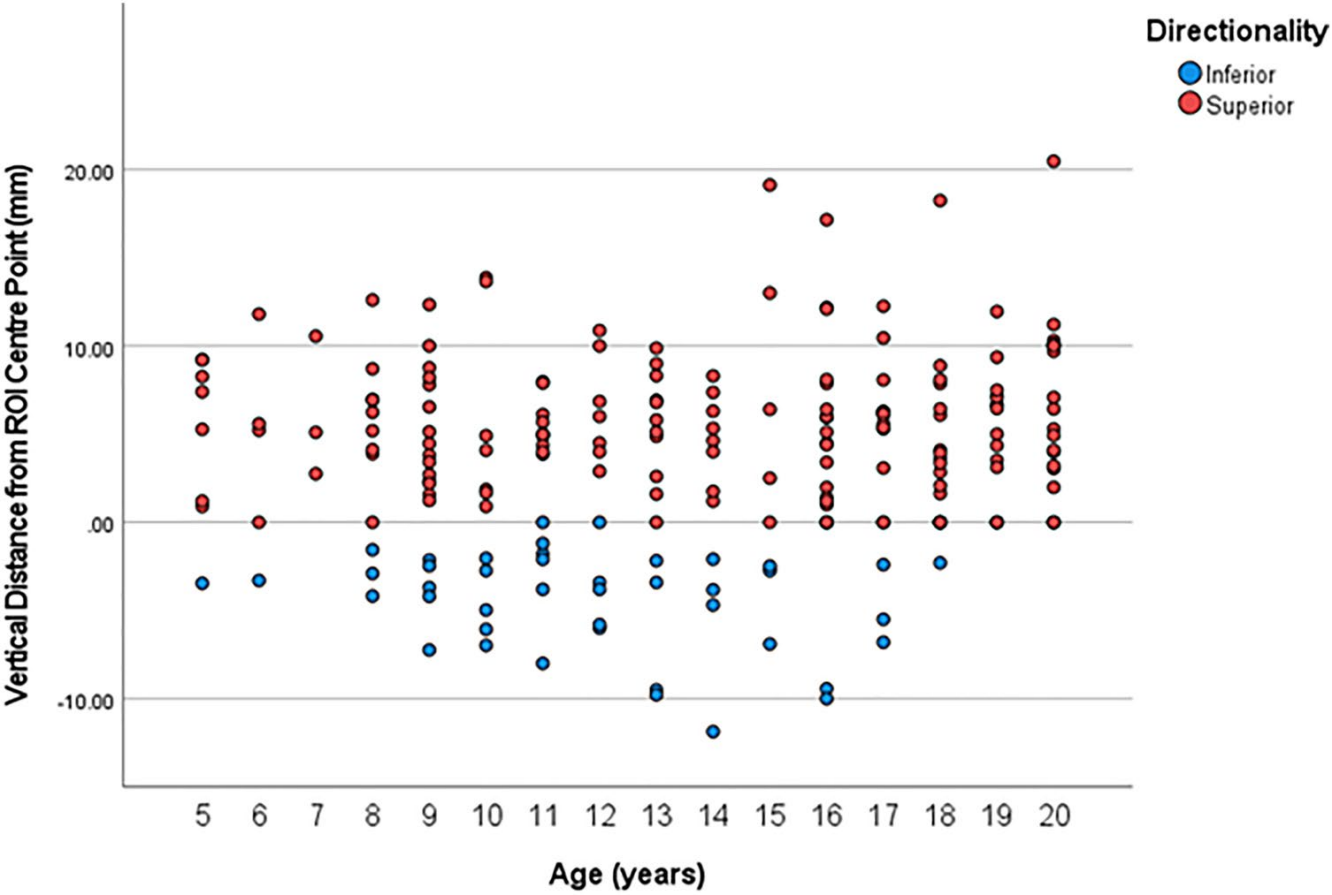
Scatterplot for the vertical distance (mm) from the ‘Jaworski point’ to our ROI centre point, with the red dots (above the 0.00 mm horizontal line) being superior and the blue dots (below the 0.00 mm horizontal line) being inferior in each age category. Noting that all samples under the age of 5 years (2 to 5 years) were pooled due to low sample numbers, with sex also being pooled due to no significant difference being observed

An independent samples T-test demonstrated no significant difference between males and females for either vertical or horizontal distances between the ‘Jaworski point’ and the ROI centre point identified in this study (*p* = 0.06 and *p* = 0.52, respectively), therefore sex was pooled. A one-way ANOVA with a Tukey post-hoc test demonstrated that the vertical distance did not change significantly with age, however there was a significant increase in the horizontal distance with age (*p* < 0.01). Using the Tukey test, neighbouring non-significantly different age categories were pooled in the following manner: 5–15 years, and 16–19 years. For vertical distances, negative values were provided to points that lied inferior to the ROI centre point, and positive values to points that lied superior to the ROI centre point. The vertical distance from the ‘Jaworski point’ to our ROI centre point demonstrated a minimum and maximum distance of − 11.87 mm and 20.47 mm, with a standard deviation of 5.52 mm, and variance of 30.56 mm as seen in Fig. 5. The horizontal distance between the two points had a minimum and maximum distance of 0.00 mm and 11.28 mm, with a standard deviation of 2.85 mm, and variance of 8.16 mm. Compared to the centre point of the ROI, the ‘Jaworski points’ were positioned anteroinferior in 21% of our samples and the remaining 79% were positioned anterosuperior. The known method resulted in 10.6% of the ‘Jaworski points’ residing outside of the maximum ROI established in this study, and 7.4% of ‘Jaworski points’ lied within 10 mm of the ROI border.

## Discussion

The reliability of bone quality assessment using quantitative ultrasound on the calcaneus in children is compromised by current systems using fixed transducers relative to a footplate and 25 mm diameter ultrasound transducers, which are too large for subadult use. These factors may result in inappropriate alignment with the anatomical ROI, and inclusion of soft tissue structures or cortical bone edges when measuring children with smaller calcanei [2, 11, 24].

Jaworski et al. (1995) have adapted current commercial devices for paediatric use by applying pads to accommodate the smaller feet of children. Although these limitations can be minimised by adapting devices for paediatric use, the applicability of the already established anatomical ROI identification method created by Jaworski et al. (1995) has not been validated via medical imaging. Without medical imaging it is unclear if the ‘Jaworski point’ lies within an acceptable ROI or how this identified region changes location with age as the calcaneal shape and size increases. This is the first study to use computed tomography to investigate and compare three novel anatomical ROI methods and the previously established paediatric method by Jaworski et al. (1995) in a subadult population, to make recommendations for improved transducer placement.

Our results recommend that clinicians use ½ inch (12.7 mm) or ¾ inch (19 mm) transducers in children younger than 7 years, with children 2–7 years having a mean ROI_max_ diameter of 24 mm. Furthermore, if measuring a child up to 2 years of age, a ½ inch (12.7 mm) diameter receiver transducer should be used as the minimum ROI diameter was 17 mm, and a ¾ inch (19 mm) transducer may result in the inclusion of surrounding soft tissue. An alternative method if clinicians only have access to one inch transducers is to occlude/minimise the ultrasound beam to a maximum of 19 mm using a circular ring or donut [9]. At 7 years of age, the mean ROI diameter increases for both males and females and it is our recommendation that any size transducer ranging from ½ inch (12.7 mm) to one inch (25.4 mm) is acceptable to use in children older than 7 years.

**Fig. 6:**
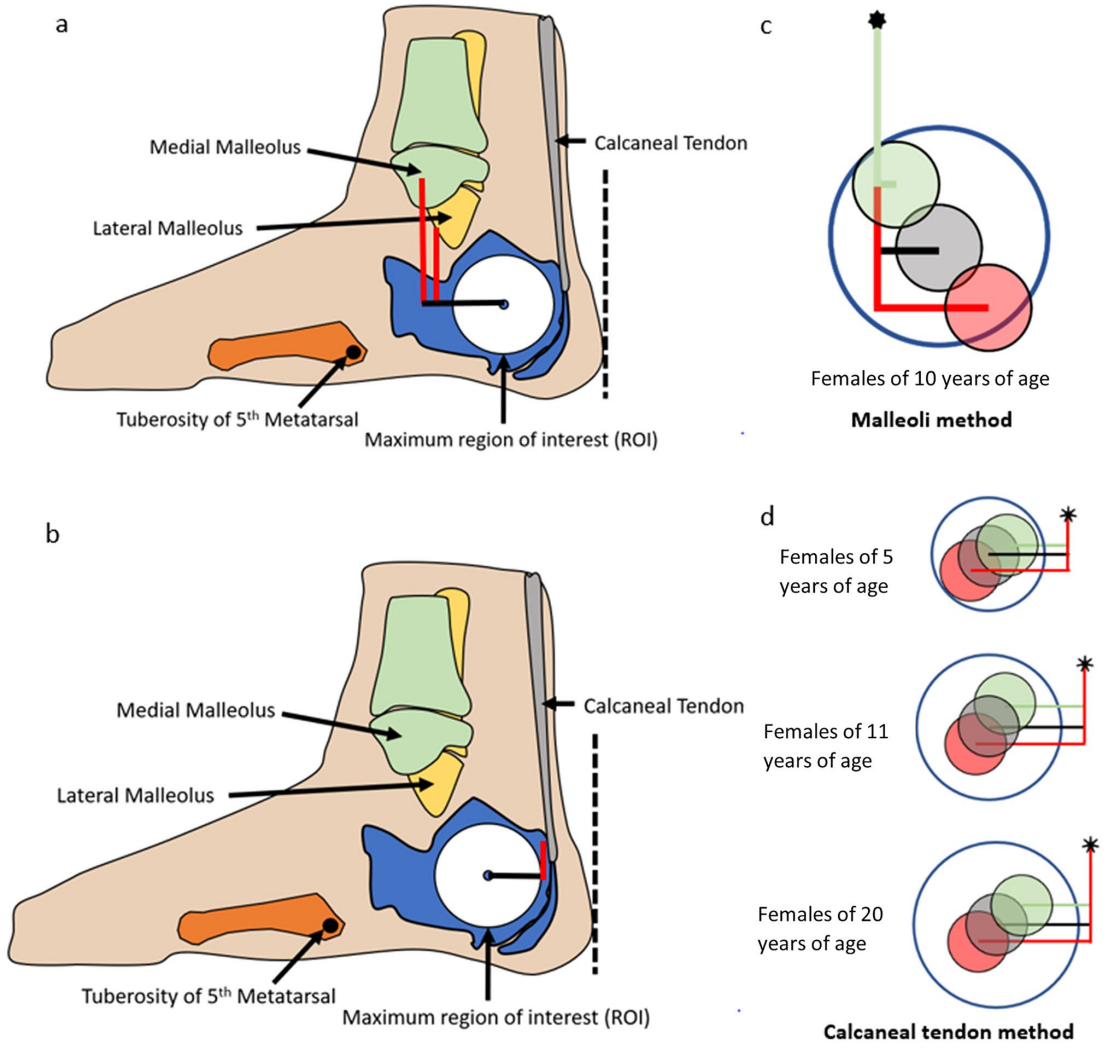
Illustration of the application and reliability of each method in a clinical setting to identify the transducer placement for QUS application. The centre point of the region of interest (ROI, white circle) is identified on the calcaneus (blue bone) using the malleoli (**a**) or calcaneal tendon (**b**) method, where the vertical (red lines) and horizontal (black lines) distances are measured from these palpable landmarks to estimate where the transducer should be positioned for optimal QUS assessment. Transducer placement relative to the ROI (blue circle) is demonstrated in (**c**) and **d** where the end of each horizontal distance measurement has a corresponding coloured ½ inch (12.4 mm) diameter ultrasound transducer according to the minimum (green lines), mean (black lines), and maximum (red lines) distances of the age range indicated. **c** Application of malleoli method in 10-year-old females; maximum distances result in transducer overlap with border of ROI. **d** Application of calcaneal tendon method in female individuals aged 5, 11, and 20 years of age. Variation between individuals of the same chronological age and sex (or within the sample measured) suggests increased risks of artefacts or undesired soft tissue interaction when applying the malleoli method to QUS (**c**), compared to the calcaneal tendon method (**d**) in which the transducers fit within the region of interest border in all cases

This study demonstrated that of the three novel anatomical ROI methods using palpable landmarks, the calcaneal tendon method was the most appropriate for clinical use to identify the preferred anatomical position of the ultrasound transducer for accurate bone assessment in subadults. The calcaneal tendon method demonstrated relatively small variance in distance measurements for both the vertical and horizontal distances in any given age range, as seen in Fig. 6, while the malleoli methods demonstrated large variation which sometimes placed the transducer outside of the maximum ROI. Although the regression models can be used to estimate the vertical and horizontal distances for individuals based on their sex and age, a descriptive table has been created for the calcaneal insertion method which allows easy clinical application; see Table 1. We would like to acknowledge that due to the retrospective design of this study that variables that may influence bone development such as sexual maturation status were not available. However, as seen in Table 1, the change in sex-sepecific distance measurements are relatively small after 7 years of age, which suggests that pubertal status is unlikely to significantly impact ROI selection.

Current clinical devices have a calf support structure which helps keep the foot in a relatively constant and slightly plantarflexed position for assessment [2]. The effects of flexion and inversion of the talocrural and subtalar joints on distance measurements from palpable landmarks were investigated in this study using 103 individuals who had varying degress of plantarflexion (20% of sample population). We found that flexion was significant in all regression models (malleoli and tendon methods), whilst inversion of the foot was only found to significantly affect the horizontal distances for both malleoli methods, and the vertical distances for the lateral malleolus method. This suggests that foot position, including whether the foot is plantar- or dorsi-flexed or inverted or everted can significantly affect the selection of the anatomical region of interest from palpable landmarks and therefore the accuracy of QUS mesurements on the calcaneus. From these findings, it is recommended that individuals be in a non-weight bearing and neural position to improve reliability of transducer placement estimation.

**Fig. 7:**
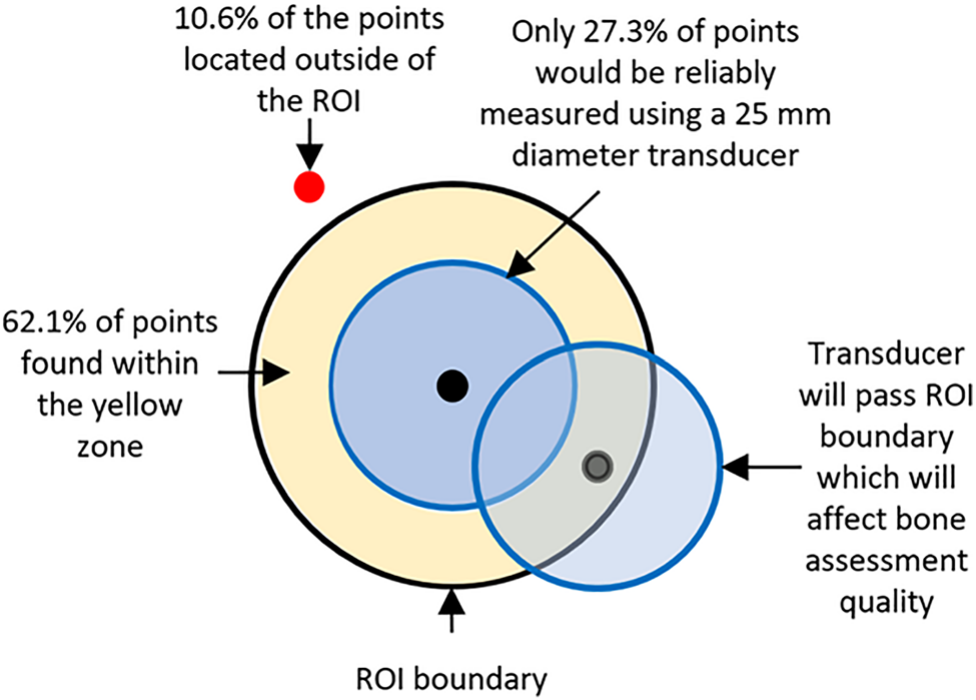
Schematic demonstrating location of ‘Jaworski points’ within the maximum ROI for all samples 2 to 20 years of age, with reference to a 25 mm (1 inch) diameter transducer. The maximum ROI is indicated by the solid black line (ROI boundary), with the solid black dot representing the ROI centre point. Based on the relative size of a 25 mm transducer (solid blue circle) only 27.3% of ‘Jaworski points’ identified using the Jaworski method would result in no overlap of the transducer with the ROI boundary. Due to high variation in ‘Jaworski point’ location, 62.1% of points are located within the yellow zone, with an example ‘Jaworski point’ (transparent black dot) being shown. The proximity of this example ‘Jaworski point’ to the ROI boundary demonstrates that when a 25 mm transducer (transparent blue circle) is used, it will lead to the undesired inclusion of surrounding soft tissue and could affect the quality of bone assessment. Out of the 62.1% of points, 26.9% of those points would reliably be measured with a 12.7 mm (1/2 inch) transducer, 27.8% of points would reliably be measured with a 19 mm (3/4 inch) transducer, and 7.4% would be measured with a 10 mm transducer. In addition, 10.6% of points were located outside of the ROI boundary where quality of bone assessment would be negatively affected

The established Jaworski et al. (1995) ROI method uses a fixed ratio for region selection regardless of age and sex. In this study we demonstrated that the majority of the ‘Jaworski points’ were located anterosuperior to the novel ROI centre point, which is the most suitable location for ultrasound transducer placement that will avoid soft tissues or edge artifacts. Both the vertical and horizontal distances from the ‘Jaworski point’ to our ROI centre point demonstrated no sex differences and large measurement ranges with 10.6% of points located outside the maximum ROI, and 7.4% lying close or on the ROI border, which in some cases were close to surrounding soft tissue structures. Only the horizontal distance between the two points demonstrated a general increase with age which may indicate an increased error as age increases. The maximum theoretical transducer diameter was also measured for each ‘Jaworski point’. The ‘Jaworski point’ maximum diameter varied dramatically across all age ranges, with 35% of the ‘Jaworski points’ lying ½ inch (12.7 mm) or closer to the ROI border as seen in Fig. 7. The relative position of the ‘Jaworski points’ are extremely variable within each age category and across all ages, which suggests that this method is not the most reliable to identify an appropriate region of interest centre point for bone tissue assessment. Furthermore, in cases when this method is used a ½ inch transducer is recommended as the 1 inch transducer may result in inclusion of soft tissue.

It can be concluded that our results recommend the calcaneal tendon method is the most reliable method compared to the novel malleoli methods and established Jaworski et al. (1995) method for the identification of an ROI centre point for ultrasound transducer placement in subadult populations due to its relatively low measurement variance compared to other methods. The application of the calcaneal tendon methodology in the measurement of QUS in a clinical setting will be investigated in future studies. Therefore, the following recommendations will result in improved ultrasound transducer placement in a subadult population:

1. Application of sex and age-specific distance measurements for the calcaneal insertion method, for individuals aged 2–20 years (Table 1)
2. ½ inch (12.7 mm) or ¾ inch (19 mm) receiver transducers should be used on individuals aged less than 7 years of age. One inch (25.4 mm) receiver transducers can be used in all individuals older than 7 years
3. Individuals be seated in a chair with the foot placed in a neutral and non-weight bearing position to maintain consistent flexion and inversion of the foot
